# Differentiation of human umbilical cord Wharton’s jelly-derived mesenchymal stem cells into endometrial cells

**DOI:** 10.1186/s13287-017-0700-5

**Published:** 2017-11-02

**Authors:** Qin Shi, JingWei Gao, Yao Jiang, Baolan Sun, Wei Lu, Min Su, Yunzhao Xu, Xiaoqing Yang, Yuquan Zhang

**Affiliations:** 1 Department of Obstetrics and Gynecology, Affiliated Hospital of Nantong University, Nantong, People’s Republic of China; 2 Department of Obstetrics and Gynecology, Suzhou Municipal Hospital, Soochow, People’s Republic of China; 3 Department of Obstetrics and Gynecology, The Second Affiliated Hospital of Soochow University, Soochow, People’s Republic of China; 4 Department of Pediatrics, Affiliated Hospital of Nantong University, Nantong, People’s Republic of China; 5 Department of Hematology, Affiliated Hospital of Nantong University, Nantong, People’s Republic of China; 6 Department of Obstetrics and Gynecology, Affiliated Hospital of Nantong University School of Medicine, 19 Xishi Road, Nantong, Jiangsu 226006 People’s Republic of China

**Keywords:** Wharton’s jelly-derived mesenchymal stem cells, Differentiation, Endometrial stromal cells, Endometrial glandular epithelial cells

## Abstract

**Background:**

Wharton’s jelly-derived mesenchymal stem cells (WJ-MSCs) are a novel and promising strategy for tissue engineering because of their ability to differentiate into many cell types. We characterized the differentiation of WJ-MSCs into endometrial epithelial cell (EEC)-like and endometrial stromal cell (ESC)-like cells and assessed the effect of 17β-estradiol and 8-Br-cAMP on the differentiation system.

**Methods:**

WJ-MSCs were treated in two ways to differentiate into EEC-like and ESC-like cells respectively: cocultured with ESCs in control/differentiation medium (17β-estradiol, growth factors); and cultured in control/differentiation medium (8-Br-cAMP alone or 8-Br-cAMP plus 17β-estrogen and growth factors). Three signaling pathway inhibitors (SB203580, PD98059, H89) were used to investigate the mechanism of WJ-MSC differentiation into ESC-like cells. Immunofluorescence, western blot and flow cytometry analyses were used to analyze expression of epithelial markers and stromal cell markers. Enzyme-linked immunosorbent assays were used to test the production of secretory proteins associated with the differentiation of ESC-like cells.

**Results:**

17β-estradiol at 1 μM downregulated vimentin and CD13 and upregulated cytokeratin and CD9 proteins, promoting the differentiation of WJ-MSCs into EEC-like cells in the coculture system. 8-Br-cAMP at 0.5 mM upregulated vimentin and CD13 and downregulated CK and CD9, promoting the differentiation of WJ-MSCs into ESC-like cells. Prolactin (PRL) and insulin-like growth factor-binding protein 1 (IGFBP1) were upregulated and the protein kinase A (PKA) signaling pathway was activated, whereas extracellular signal-regulated (ERK)1/2 and p38 mitogen-activated protein kinase (MAPK) were not affected.

**Conclusions:**

17β-estradiol at 1 μM is a good inducer for facilitating the differentiation of WJ-MSCs into EEC-like cells. 8-Br-cAMP plus estrogen and growth factors can induce the differentiation of WJ-MSCs into ESC-like cells. During the differentiation of WJ-MSCs into ESC-like cells, PRL and IGFBP1 were upregulated by the treatment and the PKA signaling pathway was activated, whereas ERK1/2 and p38 MAPK were not affected. These findings suggest a promising approach to the treatment of endometrial damage and other endometrial diseases and suggest new applications for WJ-MSCs in clinical practice.

**Electronic supplementary material:**

The online version of this article (doi:10.1186/s13287-017-0700-5) contains supplementary material, which is available to authorized users.

## Background

The human endometrium is a unique tissue that undergoes a steroid-induced monthly cycle of proliferation (regeneration during the follicular phase), differentiation (in the luteal phase), and shedding (menstruation) [1]. During the menstrual period, the functional layer of the endometrium is shed in primates and then undergoes repair during the proliferative phase to form the new layer of glandular epithelium in preparation for the new menstrual cycle [1]. An adequate endometrial thickness plays a key role in implantation of the embryo and achievement of pregnancy [2]. Damage to the endometrium associated with thinning of the lining at the time of ovulation can lead to pregnancy failure [3]. Despite the identification of certain factors that affect the growth of the endometrium, there are few agents that can ameliorate damage to the endometrium [4], and the mechanism underlying endometrial damage remains unclear.

Stem cell therapy is a novel and promising strategy with the potential to be more effective than single-agent drug therapies for many diseases [5–9]. Stem cells function in the repair of injured tissues in two ways: through the secretion of related cytokines [7], or by differentiating into the cell type of the site of injury to exert its original function [8]. Human umbilical cord Wharton’s jelly mesenchymal stem cells (WJ-MSCs) are characterized by rapid proliferation, stable biological properties, low immunogenicity, and ease of isolation [10]. In addition, WJ-MSCs can secrete a large number of cytokines and have no tumorigenic effects [11, 12]. WJ-MSCs have stronger proliferation, differentiation, and migration abilities than bone marrow MSCs, and they are ideal seed cells for cell transplantation and tissue engineering for organ replacement [10]. Previous studies showed that stem cells isolated from Wharton’s jelly have the ability to differentiate into adipocytes, osteocytes, chondrocytes, neurons, and oligodendrocytes [10]. However, their potential to differentiate into endometrial cells has not been investigated to date. Our previous study showed that WJ-MSCs can ameliorate damage to human endometrial stromal cells (ESCs) by secreting vascular endothelial growth factor (VEGF) [13]. However, whether WJ-MSCs have the potential to differentiate into endometrial cells and contribute to the repair of endometrial damage or endometrial cell renewal remains undefined.

In this study, we first explored the potential of WJ-MSCs to differentiate into endometrial epithelial cell (EEC)-like and ESC-like cells by establishing differentiation induction systems. EEC-like and ESC-like cells were identified by evaluating endometrial cell morphology, markers, and function. Analysis of the underlying mechanism revealed the involvement of the protein kinase A (PKA) signaling pathway rather than the p38 mitogen-activated protein kinase (MAPK) and extracellular signal-regulated (ERK)-1/2 signaling pathways, which are involved in the differentiation of WJ-MSCs into ESC-like cells.

## Methods

### WJ-MSC isolation, culture, and identification

Umbilical cords were collected from healthy full-term deliveries. The collection and use of human biological specimens were approved by the Ethics Committee of the Affiliated Hospital of Nantong University. The samples were incubated in serum-free Dulbecco’s Modified Eagle Medium F12 (DMEM/F-12; Hyclone, Logan, UT, USA; https://promo.gelifesciences.com/gl/hyclone/products/media.html) and transferred to the laboratory immediately. After washing, cord samples were cut into 2–3 cm sections, the umbilical vessels were removed, and Wharton’s jelly was collected and minced into pieces. The pieces were plated in tissue culture flasks containing DMEM/F-12 medium supplemented with 10% fetal bovine serum (FBS; Gibco, Waltham, MA, USA; http://www.thermofisher.com) and incubated at 37 °C in a humidified atmosphere with 5% CO_2_.

The antibodies used to identify WJ-MSCs were anti-CD14-FITC (ab28061), anti-CD45-FITC (ab27287), anti-CD79a-PE (ab155344), anti-CD90-FITC (ab11155), anti-CD34-PE (ab157304), anti-CD105-PE (ab91138), and anti-HLA-DR-FITC (ab1182), all from Abcam (Cambridge, UK; http://www.abcam.cn). Labeled cells were washed and analyzed by flow cytometry (FACS Calibur; BD Biosciences, USA; http://www.bdbiosciences.com/cn/home).

When the cells of passage 2 (P2) reached 80% confluence, they were place in osteogenesis differentiation medium (A1007201; Invitrogen, Waltham, MA, USA; http://www.thermofisher.com) or adipogenesis differentiation medium (A1007001; Invitrogen). The medium was changed every 3 days. On days 10 and 14, Alizarin red staining and Oil red O staining were performed, respectively, to identify osteoblasts and adipocytes.

### Endometrial cell isolation, culture, and identification

Endometrium samples were obtained from the Affiliated Hospital of Nantong University. Patients who suffered from intramural or pedunculated subserosal hysteromyoma without endometrial abnormalities were qualifying candidates. None of the women had taken medication or received hormonal therapy for at least 6 months prior to undergoing hysterectomy. All samples were obtained after the patients provided signed informed consent and procedures were approved by the Ethics Committee of the Affiliated Hospital of Nantong University. Collected endometrium samples were washed in sterile PBS several times to remove red blood cells, cut into small pieces (approximately 1 mm^3^), digested with 0.1% type I collagenase (C0130; Sigma-Aldrich, St Louis, MO, USA; http://www.sigmaaldrich.com) at 37 °C for 1 h, and the resulting cell suspension was filtered through sieves of two different pore sizes, firstly 100 μm and then 40 μm (100 μm Cell Strainer, 40 μm Cell Strainer, #431752, #431750; Corning, NY, USA; https://www.corning.com/cn/zh/products/life-sciences.html) to separate ESCs and EECs. The ESCs and ECCs were seeded into culture flasks containing DMEM-F12 medium supplemented with 10% FBS. EECs and ESCs were then stained with anti-cytokeratin and anti-vimentin antibodies, respectively, and identified by immunofluorescence.

### Differentiation of WJ-MSCs into EEC-like cells by coculture system

P2 WJ-MSCs and ESCs were cocultured in a Transwell system (24 mm Transwell with a 0.4-μm pore polycarbonate membrane insert, #3412; Corning). In brief, WJ-MSCs were cultured in the bottom of a six-well plate at a density of 6 × 10^5^ cells/well and ESCs were seeded on the Transwell membrane at a density of 6 × 10^4^ cells/well to separate the cells but allow soluble factors to pass freely between them. To observe the effect of endogenous factors (secreted by ESCs) and exogenous factors (estrogen and growth factors) on epithelial differentiation of WJ-MSCs, three experimental groups were set up. In Group a (control group), WJ-MSCs were cultured both in the bottom and on the membrane of the coculture system in control medium (DMEM/F12 with 2% FBS); in Group b, WJ-MSCs were cocultured with ESCs in control medium; and in Group c, WJ-MSCs were cocultured with ESCs in differentiation medium consisting of DMEM/F12 with 2% FBS, 1 × 10^–7^ mol/L 17β-estradiol (E2) (E2758; Sigma-Aldrich), 10 ng/ml epidermal growth factor (EGF) (100-47; Peprotech, USA; https://www.peprotech.com), 10 ng/ml transforming growth factor-β1 (TGF-β1) (100-21; Peprotech), and 10 ng/ml platelet-derived growth factor-BB (PDGF-BB) (100-14B; Peprotech). The medium was changed every 2 days and cultures were continued for 21 days.

Four groups treated with different estrogen levels in the coculture system were established to explore the effect of E2 on epithelial differentiation of WJ-MSCs. In Group a (control group), WJ-MSCs were cultured both in the bottom and on the membrane and fed with control medium (DMEM/F12 with 2% FBS). In Groups b, c, and d, WJ-MSCs were cocultured with ESCs and different concentrations of E2 (1 × 10^–8^, 1 × 10^–7^, or 1 × 10^–6^ mol/L respectively) in differentiation medium. The medium was changed every 2 days and cultures were continued for 21 days.

### Differentiation of WJ-MSCs into ESC-like cells by conditioned medium

P2 WJ-MSCs were cultured in the bottom of a six-well plate at a density of 6 × 10^5^ cells/well and were serum-starved overnight before culture in low-serum medium (DMEM/F12 with 2% FBS) with the following treatments: Group a, control group; Group b, 0.5 mM 8-Br-CAMP (B5386; Sigma-Aldrich) plus 10 nM E2, 10 ng/ml EGF, 10 ng/ml TGF-β1, and 10 ng/ml PDGF-BB; Group c, 0.5 mM 8-Br-CAMP; and Group d, 10 nM E2, 10 ng/ml EGF, 10 ng/ml TGF-β1, and 10 ng/ml PDGF-BB. The medium was changed every 3 days and cultures were continued for 21 days.

### Immunofluorescence staining to identify the primary cultured EECs and ESCs

Antibodies against vimentin (anti-vimentin) and CD13 (anti-CD13), which are markers of the stromal lineage, and against cytokeratin (anti-cytokeratin) and CD9 (anti-CD9), which are markers of epithelial cell lineages, were stained to identify WJ-MSC differentiation into EECs and ESCs. In brief, cells were fixed with 4% paraformaldehyde and blocked with 1% BSA. They were then incubated at 4 °C overnight with rabbit monoclonal anti-vimentin antibody (ab92547; Abcam) and mouse monoclonal anti-CD13 antibody (ab7417; Abcam) or with rabbit monoclonal anti-cytokeratin antibody (ab76126; Abcam) and mouse monoclonal anti-CD9 antibody (ab2215; Abcam). The next day, cells were stained with goat anti-rabbit-FITC (ab6717; Abcam) and goat anti-mouse-PE (ab5889; Abcam). Nuclei were counterstained with Hoechst (Beyotime Institute of Biotechnology, Jiangsu, China; https://www.beyotime.com). Stained cells were observed under a fluorescence microscope (Olympus, Tokyo, Japan).

### Western blot analysis

Proteins were resolved by SDS-PAGE and transferred onto nitrocellulose membranes. The membranes were blocked for 1 h with 5% nonfat dried milk, and then incubated at 4 °C overnight with rabbit monoclonal anti-vimentin antibody (ab92547; Abcam) or rabbit monoclonal anti-cytokeratin antibody (ab76126; Abcam) or with mouse monoclonal anti-CD13 antibody (ab7417; Abcam) or mouse monoclonal anti-GAPDH antibody (ab8245; Abcam). Primary antibodies were removed by three washes (10 min each) with Tris-buffered saline with 0.1% Tween20 (TBST) the next day. The secondary antibodies were goat anti-rabbit IgG H&L (ab6721; Abcam) or goat anti-mouse IgG H&L (ab6789; Abcam) for 2 h at room temperature. Immunoreactivity was visualized by enhanced chemiluminescence using an Alpha Innotech Imaging System (Protein Simple, Santa Clara, CA, USA). Protein band intensity was normalized to the level of GAPDH. Each experiment was repeated at least three times.

### Enzyme-linked immunosorbent assay

Protein concentrations of prolactin (PRL) and insulin-like growth factor-binding protein 1 (IGFBP1) were detected by enzyme-linked immunosorbent assay (ELISA), according to the manufacturer’s instructions (SEA846Hu, SEA052Hu; USCN, USA; http://www.uscnk.com). Minimum detection limits for the PRL and IGFBP1 assays were 0.65 and 0.065 ng/ml, respectively.

### Inhibition of the activation of PKA, ERK1/2, and p38 MAPK

Three different signaling pathway inhibitors were used to investigate the mechanism of WJ-MSC differentiation into ESCs. P2 WJ-MSCs were cultured in the bottom of a six-well plate at a density of 6 × 10^5^ cells/well with DMEM/F12 medium containing 10% FBS and the following treatments: Group a, no inhibition group; Group b, 10 μM SB203580 (inhibitor of the p38 MAPK pathway) (#5633; Cell Signaling Technology, USA; https://www.cst-c.com.cn); Group c, 10 μM PD98059 (inhibitor of the ERK1/2 pathway) (#9900; Cell Signaling Technology); Group d, 10 μM H89 (inhibitor of the PKA pathway) (2910; Tocris Bioscience, Bristol, UK; https://www.tocris.com); and Group e, no differentiation group. After treatment for 2 h, the media of Groups a–d were changed to low-serum medium (DMEM/F12 with 2% FBS) with 0.5 mM 8-Br-CAMP plus 10 nM E2, 10 ng/ml EGF, 10 ng/ml TGF-β1, and 10 ng/ml PDGF-BB; the medium of Group e was changed to low-serum medium (DMEM/F12 with 2% FBS) and all cultures were continued for 21 days.

### Flow cytometry

WJ-MSCs in the five groups were treated for 21 days and digested and stained with monoclonal antibodies at room temperature for 30 min before analysis by flow cytometry. The antibodies used to confirm the differentiation of WJ-MSCs into ESC-like and EEC-like cells were anti-vimentin-PE (ab8978; Abcam), anti-CD13-PE (ab140233; Abcam), anti-cytokeratin-Alexa Fluor 647 (ab192468; Abcam), and anti-CD9-FITC (ab34162; Abcam). Labeled cells were washed and analyzed by flow cytometry (BriCyte E6; Mindray, China; http://www.mindray.com/cn/index.html).

### Statistical analysis

**Fig. 1:**
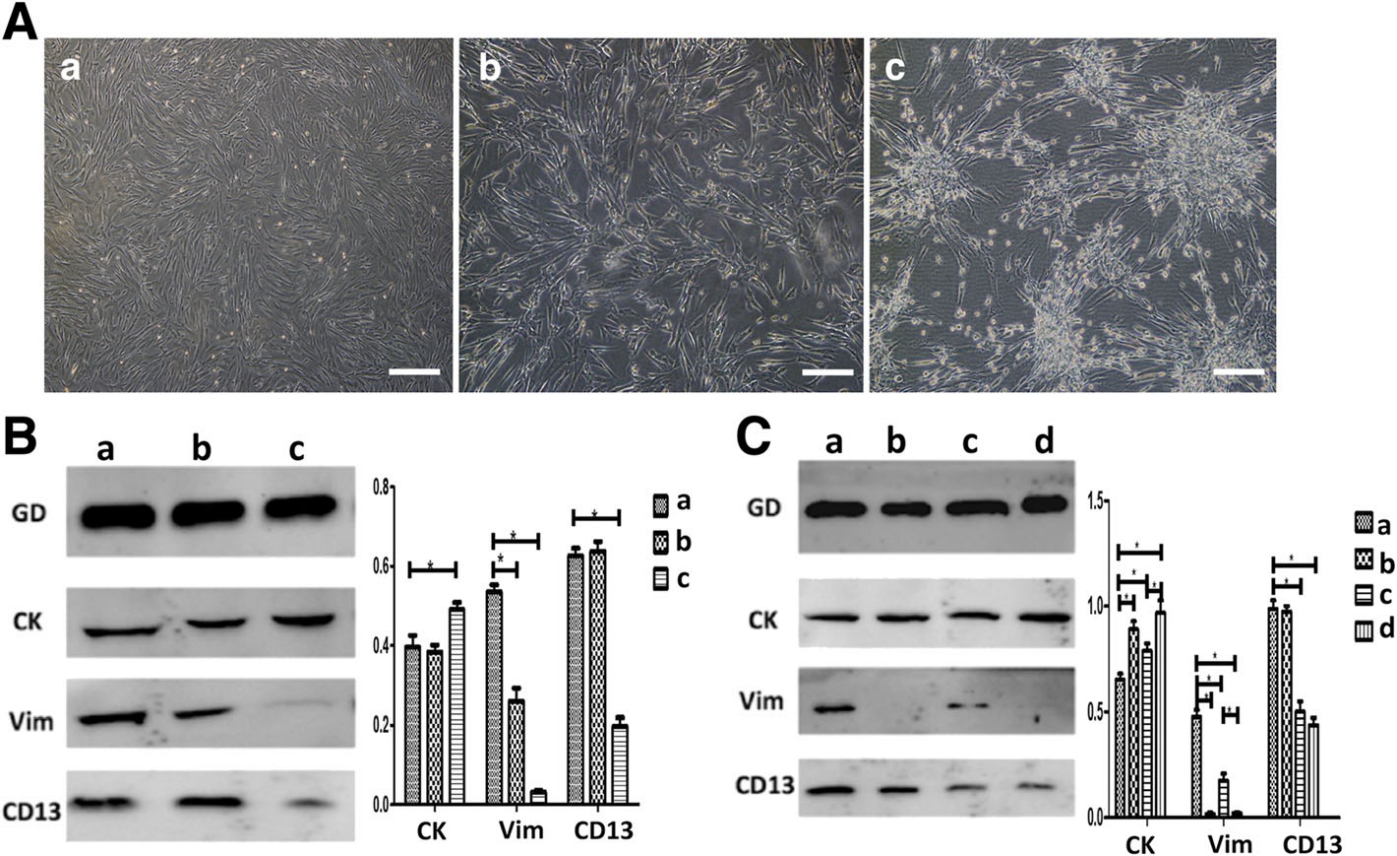
WJ-MSCs differentiate into EEC-like cells in the coculture system. (**A**) Morphologic changes of WJ-MSCs after induced differentiation in three groups: (a) WJ-MSCs cultured both in the bottom and the membrane of the coculture system in control media (DMEM/F12 with 2% FBS). (b) WJ-MSCs cocultured with ESCs in control medium; (c) WJ-MSCs cocultured with ESCs in differentiation medium (DMEM/F12 with 2% FBS, and 1 × 10^7^ mol/l 17β-E2, 10 ng/ml TGF, 10 ng/ml EGF, and 10 ng/ml PDGF-BB). Bar represents 200 μm. (**B**) Western blot analyses of cytokeratin, CD9, and vimentin in cell lysates isolated from WJ-MSCs in the three groups. Fusion proteins detected with anti-cytokeratin (CK), anti-vimentin (Vim), and anti-CD13 antibodies, and anti-GAPDH (GD) antibody was used as a loading control. Error bars represent SEM. *p < 0.05. (**C**) Western blot analyses of cytokeratin, CD9, and vimentin in cell lysates isolated from WJ-MSCs to show the effect of concentration of 17β-E2 on the differentiation of WJ-MSCs. Fusion proteins detected with anti-CK, anti-Vim, and anti-CD13 antibodies, and anti-GD antibody was used as a loading control. Group a, WJ-MSCs cultured both in the bottom and on the membrane and fed with control medium; Groups b, c, and d, WJ-MSCs cocultured with ESCs with different concentrations of 17β-E2 (1 × 10^–8^, 1 × 10^–7^, or 1 × 10^–6^ mol/L respectively) in differentiation medium. Error bars represent SEM. *p < 0.05

Data were expressed as the mean ± SD. Statistical analyses were performed using GraphPad Prism 6 software. Western blot, ELISA and flow cytometry data (Figs. 1B, C, 3C– E and 6A, B, D) were evaluated by independent-sample *t* test comparing the means between two groups, and one-way analysis of variance (ANOVA) making multiple comparison among three or more groups. Statistical *p* <0.05 was considered significant.

## Results

### WJ-MSC culture and identification

Minced Wharton’s jelly samples were incubated in tissue culture flasks, and after 6–7 days several triangular and spindle-shaped cells that had dissociated from the tissues were detected. The adherent cells reached 80% confluence after 1 week. Cells at P2 exhibited a spindle shape, and upon reaching confluence formed a whirlpool-like pattern on the flask surface (Additional file 1: Figure S1A).

Flow cytometry results indicated that WJ-MSCs had a characteristic MSC phenotype. They were negative for CD14, CD34, CD45, CD79a, and HLA-DR, and positive for CD105 and CD90 (Additional file 1: Figure S1B).

The differentiation potential of WJ-MSCs toward the osteogenic and adipogenic lineages was tested as described. Cells cultured in osteogenic induction medium showed alkaline phosphatase activity, whereas cells cultured in adipogenic induction medium accumulated lipid droplets in the cytoplasm (Additional file 1: Figure S1C). No lipid vacuoles or mineralized matrix formation was observed in WJ-MSCs maintained in regular growth medium (Additional file 1: Figure S1C).

### Culture and identification of endometrial cells

P1 ESCs were spindle-shaped fibroblast-like cells (Additional file 2: Figure S2A) and P1 EECs were polygonal cells rounder than stromal cells (Additional file 2: Figure S2B). Cultured ESCs and EECs were identified by immunofluorescence staining, which showed that ESCs were positive for the stromal cell markers vimentin (Vim) and CD13, and negative for the epithelial cell markers cytokeratin (CK) and CD9 (Additional file 2: Figure S2C). By contrast, EECs were positive for CK and CD9, and negative for Vim and CD13 (Additional file 2: Figure S2D).

### Upregulation of CK and CD9 and downregulation of Vim and CD133 by the coculture system indicated the differentiation of WJ-MSCs into EEC-like cells

Three experimental groups were established to observe the effect of endogenous factors (secreted by ESCs) and exogenous factors (estrogen and growth factors) on the differentiation of WJ-MSCs. Cultured cells were analyzed for cell morphology and the expression of the epithelial cell markers CK and CD9 and the stromal cell markers Vim and CD13 by immunofluorescence and western blot analyses.

After approximately 21 days in culture, cells in Group c formed a mass, whereas cells in Group b showed an irregular growth pattern that differed from the spiral growth pattern observed in the control group (Group a) (Fig. 1A). The epithelial cell marker CK was remarkably upregulated, whereas the stromal cell markers Vim and CD13 were downregulated in Group c as determined by western blot analysis. No significant difference was observed between the control group (Group a) and Group b (Fig. 1B).

**Fig. 2:**
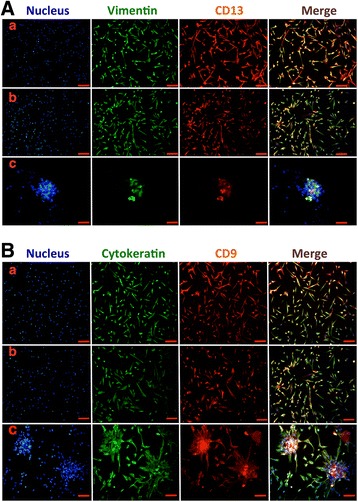
Immunofluorescent staining of WJ-MSC differentiation into EEC-like cells in the coculture system. (**A**) Cells in the three groups stained with anti-vimentin antibodies (green), anti-CD13 (red), and the Hoechst nuclear dye (blue). Merged images displayed in the fourth column. (**B**) Cells in the three groups stained with anti-cytokeratin antibodies (green), anti-CD9 (red), and the Hoechst nuclear dye (blue). Group a, WJ-MSCs cultured both in the bottom and the membrane of the coculture system in control media: WJ-MSCs showed strong positive staining for vimentin, CD13, cytokeratin, and CD9. Group b, WJ-MSCs cocultured with ESCs in control medium: cells still positively stained by vimentin, CD13, cytokeratin, and CD9. Group c, WJ-MSCs cocultured with ESCs in the differentiation medium: cells strongly positively stained by cytokeratin and CD9 but weakly stained by vimentin and CD13. Bar represents 200 μm

Immunofluorescence analysis showed similar results. Undifferentiated WJ-MSCs in Groups a and b were positive for CK, CD9, Vim, and CD13, whereas WJ-MSCs in Group c were mildly positive for Vim and CD13 and strongly positive for CK and CD9 (Fig. 2).

These results suggest that only the joint action of endogenous factors (secreted by ESCs) and exogenous factors (estrogen and growth factors) can induce the differentiation of WJ-MSCs into EEC-like cells. As estrogen has a strong effect on a woman’s menstrual cycle, in the next experiment we further investigated the role of E2 in the differentiation of WJ-MSCs into EEC-like cells.

### Determination of the optimal concentration of E2 for inducing the differentiation of WJ-MSCs into EEC-like cells in the coculture system

To explore the effect of E2 on epithelial differentiation of WJ-MSCs, four groups were established and treated with different doses of estrogen in the coculture system. In Group a, WJ-MSCs were cultured both in the bottom and on the membrane in control medium (DMEM/F12 with 2% FBS). In Groups b, c, and d, WJ-MSCs were cocultured with ESCs and different concentrations of E2 (1 × 10^–8^, 1 × 10^–7^, and 1 × 10^–6^ mol/L respectively) in differentiation medium. Cells were cultured for 21 days, and the epithelial cell marker CK and the stromal cell markers Vim and CD13 were detected by western blot analysis. The results showed that the expression levels of CK were highest and those of Vim and CD13 were lowest in cells cultured with 1 × 10^–6^ mol/L E2 (Group d) (Fig. 1C). This suggested that 1 × 10^–6^ mol/L E2 provided the optimal microenvironment for the differentiation of WJ-MSCs into epithelial lineages.

### Incubation of WJ-MSCs in conditioned medium upregulated Vim and CD13 and downregulated CK and CD9, inducing the differentiation of WJ-MSCs into ESC-like cells

**Fig. 3:**
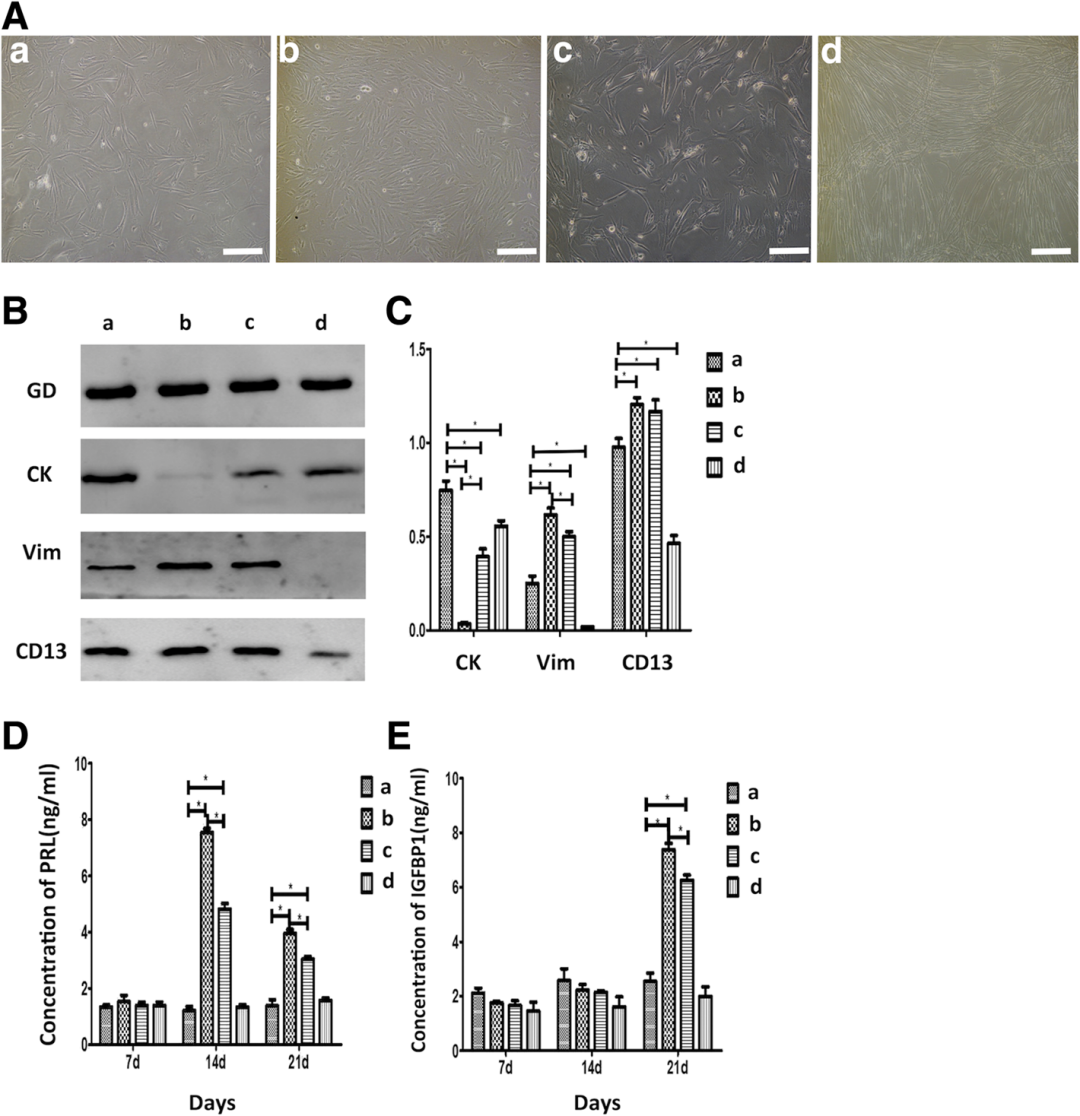
WJ-MSCs differentiate into ESC-like cells. (**A**) Morphologic changes of WJ-MSCs. (a) Control group: cells still triangular and spindle-shaped. (b) 0.5 mM 8-Br-CAMP plus 10 nM 17β-estradiol (E2) and 10 ng/ml epidermal growth factor (EGF), 10 ng/ml transforming growth factor (TGF), and 10 ng/ml platelet-derived growth factor-BB (PDGF-BB): cells became shorter and slightly rounded at both ends. They become smaller and the cytoplasm was reduced. (c) 0.5 mM 8-Br-CAMP group: cells had a similar trend to Group b but less obvious. (d) 10 nM 17β-E2 and 10 ng/ml EGF, 10 ng/ml TGF, and 10 ng/ml PDGF-BB group: cells longer and narrower than the control group. They were arranged in a disordered pattern. Bar represents 200 μm. (**B**) Western blot analyses of cytokeratin, CD9, and vimentin in cell lysates isolated from WJ-MSCs in the four groups. Fusion protein detected with anti-cytokeratin (CK), anti-vimentin (Vim), and anti-CD13 antibodies, and anti-GAPDH (GD) antibody was used as a loading control. (**C**, **D**, **E**) Quantification of western blot and ELISA data representing three independent experiments. Error bars represent SEM. *p < 0.05. d days, PRL prolactin, IGFBP1 insulin-like growth factor-binding protein 1

The differentiating effect of 8-Br-CAMP was shown in previous research in BM-MSCs [14]. The present results indicated that after incubation in differentiation medium for 21 days, WJ-MSCs showed a spindle shape and resembled ESCs (Fig. 3A) in both Group b (Br-CAMP, estrogen, and growth factors group) and Group c (Br-CAMP group). To determine the optimal conditioning medium for the differentiation of WJ-MSCs into ESC-like cells, we further characterized the markers of ESCs in each group by western blot, immunofluorescence, and ELISA analyses.

**Fig. 4:**
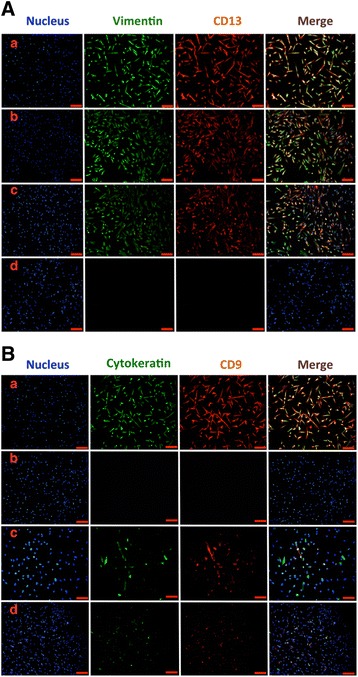
Immunofluorescence staining of WJ-MSC differentiation into ESC-like cells in the four groups: (a) untreated WJ-MSCs; (b) WJ-MSCs treated with 8-Br-CAMP and E2; (c) WJ-MSCs treated with 8-Br-CAMP; (d) WJ-MSCs treated with E2. (**A**) Cells in the four groups stained with anti-vimentin antibodies (green), anti-CD13 (red), and the Hoechst nuclear dye (blue). Merged images displayed in the fourth column. (**B**) Cells in the four groups stained with anti-cytokeratin antibodies (green), anti-CD9 (red), and the Hoechst nuclear dye (blue). Merged images displayed in the fourth column. Bar represents 200 μm

Immunofluorescence analysis showed that WJ-MSCs in Group a were positive for CK, CD9, Vim, and CD13. Compared with the control group, cells in Group b (Br-CAMP, estrogen, and growth factors group) were only positive for the stromal cell markers Vim and CD13 and negative for the epithelial markers CK and CD9. A similar trend was observed in Group c (Br-CAMP group), although weak staining for CK and CD9 was detected. All four markers were reduced in Group d (estrogen and growth factors group) (Fig. 4).

Western blot analysis showed that the stromal markers Vim and CD13 were upregulated in Groups b and c, with higher expression in Group b. The epithelial marker CK was significantly downregulated in Groups b and c, and expression in Group b was weaker than that in Group c. No stromal cell markers were detected in Group d (Fig. 3B, C).

### Incubation of WJ-MSCs in conditioned medium increased the secretion of PRL and IGFBP1

To further confirm the results of the differentiation of MSCs into ESCs, ESC-like cells were characterized by detecting the markers PRL and IGFBP1 after treatment of WJ-MSC with the four different regimens described. ELISA results showed that PRL and IGFBP1 were significantly upregulated in Groups b and c compared with the control group at the same time points (days 7, 14, and 21), with the highest expression levels observed on day 14 for PRL and day 21 for IGFBP1. In addition, a significant difference in IGFBP1 and PRL protein expression was observed between WJ-MSCs treated with 8-Br-cAMP plus estrogen and growth factors and those treated with 8-Br-cAMP alone (*p* < 0.001) (Fig. 3D, E), suggesting that differentiated WJ-MSCs had the same secretion pattern as ESCs.

### Activation of PKA but not p38 MAPK and ERK1/2 promoted WJ-MSC differentiation into ESC-like cells

**Fig. 5:**
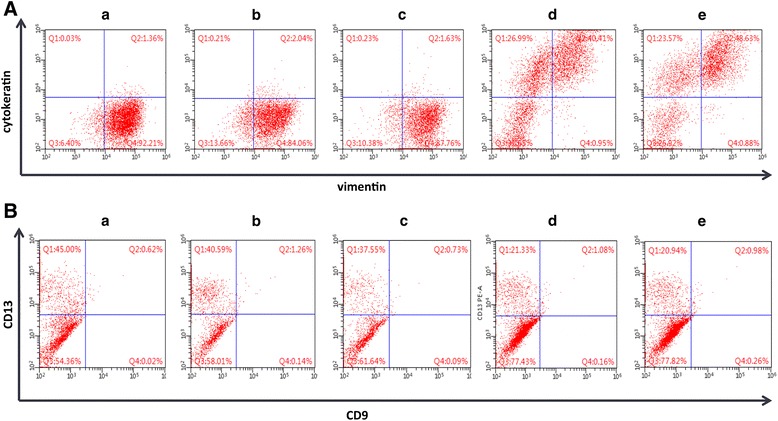
H89 blocks the 8-Br-cAMP-induced differentiation of WJ-MSCs into ESC-like cells. (**A**) Flow cytometric analysis of cells in the five groups using antibodies against vimentin and CD13. (**B**) Flow cytometric analysis of cells in the five groups using antibodies against cytokeratin and CD9. (a) no inhibition group; (b) p38 MAPK-block group; (c) ERK1/2-block group; (d) PKA-block group; (e) no differentiation group. Cells in Groups a–d treated with 8-Br-cAMP for 21 days

**Fig. 6:**
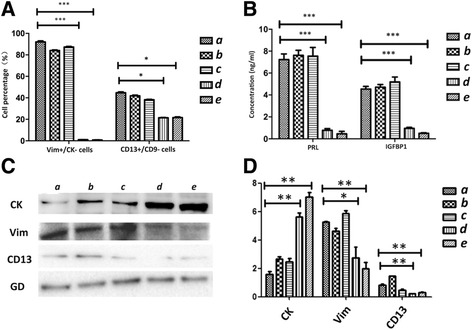
Quantification of flow cytometry, western blot, and ELISA data to investigate the impact of p38 MAPK, ERK1/2, and PKA activation on the differentiation of WJ-MSCs into ESC-like cells. (**A**) Quantification of flow cytometry data for the Vim^+^/CK^–^ cell and CD13^+^/CD9^–^ cell percentages in each group. (a) no inhibition group; (b) p38 MAPK-block group; (c) ERK1/2-block group; (d) PKA-block group; (e) no differentiation group. Cells in Groups a–d treated with 8-Br-cAMP for 21 days. Data represent results of three independent experiments. Error bars represent SEM. *p < 0.05; ***p < 0.001. (**B**) Quantification of ELISA data representing three independent experiments. Error bars represent SEM. ***p < 0.001. (**C**) Western blot analyses of cytokeratin (CK), CD9, and vimentin (Vim) in cell lysates isolated from WJ-MSCs in the five groups. Fusion protein detected with anti-CK, anti-Vim, and anti-CD13 antibodies, and anti-GAPDH (GD) antibody was used as a loading control. (**D**) Quantification of western blot data representing three independent experiments. Error bars represent SEM. *p < 0.05; **p < 0.005. PRL prolactin, IGFBP1 insulin-like growth factor-binding protein 1

According to our previous studies, WJ-MSCs have the ability to repair damaged ESCs. Our current study showed that differentiated WJ-MSCs not only express ESC markers, but also possess the secretory function of ESCs. However, the signaling pathway involved in the transformation of WJ-MSCs into ESC-like cells remained unclear. We therefore investigated the p38 MAPK, ERK1/2, and PKA pathways. Flow cytometry data showed that the percentages of Vim^+^/CK^–^ cells and CD13^+^/CD9^–^ cells in Group a (no inhibition group), Group b (SB203580 group), and Group c (PD98059 group) were significantly higher than those in Group d (H89 group) and Group e (no differentiation group) (*p* < 0.001; Figs. 5A, B and 6A). Western blot analysis revealed that, compared with the no inhibition group, the stromal markers Vim and CD13 were not substantially decreased in Groups b and c, whereas they were decreased in Groups d and e. The epithelial marker CK was significantly increased in Groups d and e, whereas it was not increased in Groups b and c (Fig. 6C, D).

ELISA showed that, compared with the no inhibition group, the levels of PRL and IGFBP1 secreted by WJ-MSCs were decreased in Groups d and e but not in Groups b and c (Fig. 6B). Taken together, these results suggested that H89 but not SB203580 and PD98059, which is a specific PKA inhibitor, blocks WJ-MSC differentiation into ESC-like cells.

## Discussion

WJ-MSCs are regarded as an ideal source of stem cells for clinical treatment and scientific research applications [10]. Several studies demonstrated that WJ-MSCs can differentiate into the cells of all three embryonic layers under specific conditions [15–18] and that they function in tissue repair or regeneration. Xu et al. [19] induced WJ-MSCs to differentiate into sweat gland-like cells (SGCs) using conditioned medium containing recombinant human keratinocyte growth factor and found that the differentiated SGCs had a potential therapeutic application in the regeneration of destroyed sweat glands and injured skin in vivo. Qu et al. [20] found that laminin 411 acts as a potent inducer of the differentiation of WJ-MSCs into insulin-producing cells (IPCs) via the Pdx1 and Ngn3 signaling pathways, and transfusion of the differentiated IPCs improved the symptoms and survival of rats with type 1 diabetes. Aghajanova et al. [14] treated bone marrow-derived MSCs with estradiol and progesterone, bone morphogenetic protein 2, and activators of the PKA pathway (8-Br-CAMP), inducing their differentiation into ESCs. However, they did not show the differentiation of bone marrow-derived MSCs into EECs and the related mechanism. Although the ability of WJ-MSCs to repair endometrial damage was reported previously [13, 21], the endometrial differentiation potential of WJ-MSCs has not been investigated to date. As WJ-MSCs have stronger proliferation, differentiation, and migration abilities than bone marrow-derived MSCs [10], we hypothesized that if they could differentiate into EECs and ESCs, WJ-MSCs could be of value for the treatment of endometrial injury. In this study, we first explored the capacity of WJ-MSCs to differentiate into endometrial-like cells (EEC-like and ESC-like cells). We successfully designed a differentiation strategy and confirmed that the differentiation medium or methods for the two cell types are not the same.

Li et al. [22] showed that WJ-MSCs incubated with rat urogenital sinus stromal cells and transplanted into the renal capsule in vivo differentiated into prostate epithelial-like cells, as verified by the expression of prostate epithelial cell-specific markers including prostate specific antigen. Their study indicated that WJ-MSCs have the capability to differentiate into epithelial-like cells, which are normally derived from the endoderm. Garzón et al. [23, 24] demonstrated the potential of WJ-MSCs to differentiate into oral mucosa and skin epithelioid cells and corneal epithelioid cells. To examine the ability of WJ-MSCs to differentiate into EEC-like cells, we used the Transwell coculture system to culture ESCs with WJ-MSCs together. In this system, cytokines can freely pass the 0.4-μm pore size carbon polyester film, whereas the cells cannot. The experimental results showed that under the combined action of growth factors, functional proteins, cell factors secreted by ESCs, and the added factors (TGF-β1, EGF, and PDGF-BB) as well as estrogen, WJ-MSCs began to grow in a mass instead of the spiral pattern observed in the control group. Furthermore, the epithelial cell markers CK and CD9 were remarkably elevated and the stromal cell markers Vim and CD13 were obviously decreased, as determined by immunofluorescence and western blot analyses. Since estrogen plays a significant role in a woman’s menstrual cycle, we further studied the influence of the concentration of estrogen on the differentiation of WJ-MSCs into EEC-like cells. The gradient concentration of E2 was 1 × 10^–8^–1 × 10^–6^ mol/L. Western blot analysis was performed to compare the expression of CK, Vim, and CD13. The results indicated that 1 × 10^–6^ mol/L E2 provided the optimum microenvironment for the differentiation of WJ-MSCs into EEC-like cells.

In the clinic, there are many diseases and disorders that result in endometrial injury, such as Asherman’s syndrome, infertility, and amenorrhea [25, 26]. Patients with these diseases often experience deep sorrow. Consequently, the study of endometrial repair after pathological damage has become increasingly important. The differentiation of MSCs into endometrial stromal-like cells may provide a new method to treat these diseases as well as to establish physiological menstruation and pregnancy in clinical practice. Aghajanova et al. [14] reported that 8-Br-CAMP is an inducible factor in the differentiation of BM-MSCs into ESCs. Our previous study confirmed that WJ-MSCs could be used to repair the damaged endometrium through the secretion of VEGF. In this study, we investigated 8-Br-cAMP as an inducer of differentiation in WJ-MSCs. Recent studies showed that the endometrium can be repaired after menstruation in ovariectomized immunodeficient mice, which suggested a reparative effect of estrogen after menstruation [27]. However, a different study showed that estrogen and progesterone receptor expression increased during the stage of endometrial regeneration [28]. In the present study, we investigated the effect of different concentrations of estrogen on promoting the differentiation of WJ-MSCs into ESC-like cells. The results showed that the stromal cell markers Vim and CD13 were remarkably elevated and the epithelial cell markers CK19 and CD9 were decreased both in the 8-Br-CAMP-treated group and the 8-Br-CAMP plus estrogen group after induction for 21 days; however, the induction was more effective in the medium including estrogen. In the 8-Br-CAMP plus estrogen group, IGFBP1 began to increase on day 14 and peaked after 21 days of induction. PRL was also significantly elevated on day 14. In addition, we found that 10 nM estrogen accelerated the differentiation, indicating that the effect was dose dependent.

The cAMP-dependent PKA pathway plays a role in promoting cell proliferation and inhibiting apoptosis [29]. H89, a cAMP/PKA signaling pathway inhibitor, can block the PKA signaling pathway [30]. The p38 MAPK pathway is involved in the process of secretion of VEGF by MSCs. SB203580, a specific inhibitor of p38 MAPK, significantly blocks the secretion of VEGF from MSCs induced by hypoxia-inducible factor 1, whereas hypoxia can induce MSCs to differentiate into vascular endothelial cells [31]. Although the hypoxia-induced secretion of VEGF is involved in the differentiation of MSCs into endothelial cells, it is unclear whether the p38 MAPK pathway plays a role in the differentiation of WJ-MSCs into endometrioid cells. Xu et al. [32] found that VEGF induces the differentiation of MSCs into vascular endothelial cells through the ERK1/2 pathway, and PD98059 blocks the VEGF-mediated differentiation of MSCs. However, there are no reports on the effects of the ERK1/2 signaling pathway on the differentiation of MSCs into endometrial cells.

Our previous studies showed that ESCs play an irreplaceable role in repairing endometrial damage; therefore, we further explored the molecular mechanisms underlying the differentiation of WJ-MSCs into ESC-like cells. We used H89 to examine the involvement of the cAMP/PKA pathway in the differentiation of WJ-MSCs into ESC-like cells. WJ-MSCs were treated with 10 μM H89 for 2 h before adding differentiation medium. Flow cytometry, western blot, and ELISA analyses were used to determine the extent of cell differentiation. We found that blocking the PKA pathway with H89 decreased the percentages of Vim^+^/CK^–^ cells and CD13^+^/CD9^–^ cells, and inhibited the differentiation of WJ-MSCs as determined by flow cytometry and western blot analysis. However, blocking the ERK1/2 and p38 MAPK pathways did not significantly affect the differentiation of WJ-MSCs into ESC-like cells, as indicated by the detection of protein/hormone contents by flow cytometry, western blot, and ELISA analyses, which were comparable to those in the control group. These results indicated that the cAMP/PKA pathway may contribute to the differentiation of WJ-MSCs into ESC-like cells, whereas the ERK1/2 and p38 MAPK pathways were not involved. The effects of the two inhibitors of ERK1/2 and p38 MAPK at different concentrations on WJ-MSCs were not assessed, and this should be investigated in future studies.

Recently, multilineage differentiating stress enduring (Muse) cells have been reported to be a novel population of nontumorigenic pluripotent stem cells [33]. They are able to differentiate into cells from all three embryonic germ layers both spontaneously and under media-specific induction. The existence of Muse cells has been demonstrated in bone marrow, skin cells, and adipose tissue. Ratajczak et al. [34, 35] found that adult tissue harbors a population of very rare stem cells, named very small embryonic-like stem cells (VSELs). VSELs are present in umbilical cord blood and bone marrow, and have biologic features including the pluripotent nature and the ability to secrete growth factors, making them attractive for cell-therapy strategies in humans [36]. The current strategy for purifying murine VSELs is based on sorting any nucleated cells that are slightly smaller than erythrocytes (4–5 mm) with round shape and express the Sca1^+^Lin^−^CD45^−^ phenotype [37]. However, there is no evidence proving that Muse cells or VSELs can be isolated from Wharton’s jelly [37]. In our study, WJ-MSCs were derived from Wharton’s jelly that was collected and minced into pieces after all the umbilical blood and vessels were removed. They were negative for the hematopoietic markers (Additional file 1: Figure S1B) and were therefore distinct from the MSCs isolated from hematopoietic organs such as umbilical cord blood and bone marrow. Moreover, we have not observed these round cells in the purified WJ-MSCs under a phase-contrast microscope. We identified WJ-MSCs rigorously both before and after the differentiation by cell morphology and surface markers, and hence concluded that such a group of cells differentiate in specific microenvironments.

## Conclusions

The present study is the first to investigate the capacity of WJ-MSCs to differentiate into EEC-like and ESC-like cells in specific microenvironments. E2 at 1 μM is a good inducer for facilitating the differentiation of WJ-MSCs into EEC-like cells. 8-Br-cAMP plus estrogen and growth factors can induce the differentiation of WJ-MSCs into ESC-like cells by activating the PKA signaling pathway, whereas it had no effect on the ERK1/2 or p38 MAPK pathway. These findings may provide a promising approach for the treatment of endometrial damage and other endometrial diseases and might increase the applications of WJ-MSCs in clinic practice.

## Additional files

Additional file 1: Figure S1. showing identification of WJ-MSCs. (**A**) Observation of WJ-MSCs under a phase-contrast microscope. (a) Seven days after the tissues of Wharton’s jelly were plated, many triangular and spindle-shaped cells dissociated from the tissues. (b) About half a month later, these adherent cells were able to reach 80% confluence. (c) Third-generation cells exhibited a spindle shape and upon reaching confluence formed a whirlpool-like pattern. Bar represents 200 μm. (**B**) Surface antigens of WJ-MSCs in flow cytometry. WJ-MSCs were positive for CD90 and CD105; WJ-MSCs were negative for CD14, CD34, CD45, CD79a, and HLA-DR. Results confirmed that cells were MSCs but nonhematopoietic. (**C**) Differentiation potential of WJ-MSCs toward osteogenic and adipogenic lineages. Osteogenic differentiation assayed using the von Kossa procedure and adipogenic differentiation determined by formation of lipid vacuoles after induction. (a) No mineralized matrix formation found in WJ-MSCs cultured in regular growth medium. (b) Osteogenic differentiation determined by staining with Alizarin red after osteogeneic induction. (c) No lipid vacuoles found in WJ-MSCs cultured in regular medium. (d) Adipogenic differentiation detected by Oil red O staining. Bar represents 400 μm

Additional file 2: Figure S2. showing identification of ESCs and EECs. (**A**) Morphological characteristics of ESCs. Bar represents 200 μm. (**B**) Morphological characteristics of EECs. Bar represents 200 μm. (**C**) Observation of ESCs after immunofluorescent staining. Results show ESCs in primary culture positively stained by vimentin and CD13 but negatively stained for cytokeratin and CD9. (a), (e) Nuclear counterstaining with Hoechst 33342. (b) ESCs positively stained by vimentin. (c) ESCs positively stained by CD13. (d) Merger of (a)–(c). (f) ESCs negatively stained by cytokeratin. (g) ESCs negatively stained by CD9. (h) Merger of (e)–(g). Bar represents 200 μm. (**D**) Observation of EECs after immunofluorescent staining. Results show that EECs in primary culture were positively stained by cytokeratin and CD9 but negatively stained for vimentin and CD13. (a), (e) Nucleal counterstaining with Hoechst 33342. (b) EECs negatively stained by vimentin. (c) EECs negatively stained by CD13. (d) Merger of (a)–(c). (f) EECs positively stained by cytokeratin. (g) ESCs positively stained by CD9. (h) Merger of (e)–(g). Bar represents 200 μm (TIFF 31403 kb)

## Abbreviations

CK: Cytokeratin
E2: 17β-estradiol
EEC: Endometrial epithelial cell
ESC: Endometrial stromal cell
IGFBP1: Insulin-like growth factor-binding protein 1
PRL: Prolactin
VEGF: Vascular endothelial growth factor
WJ-MSC: Wharton’s jelly-derived mesenchymal stem cell
