# Supplementary figures and images for: Differentiation of human umbilical cord Wharton’s jelly-derived mesenchymal stem cells into endometrial cells
Source: Stem Cell Res Ther. 2017 Nov 2;8:246. doi: 10.1186/s13287-017-0700-5 (PMC5667478; doi:10.1186/s13287-017-0700-5)

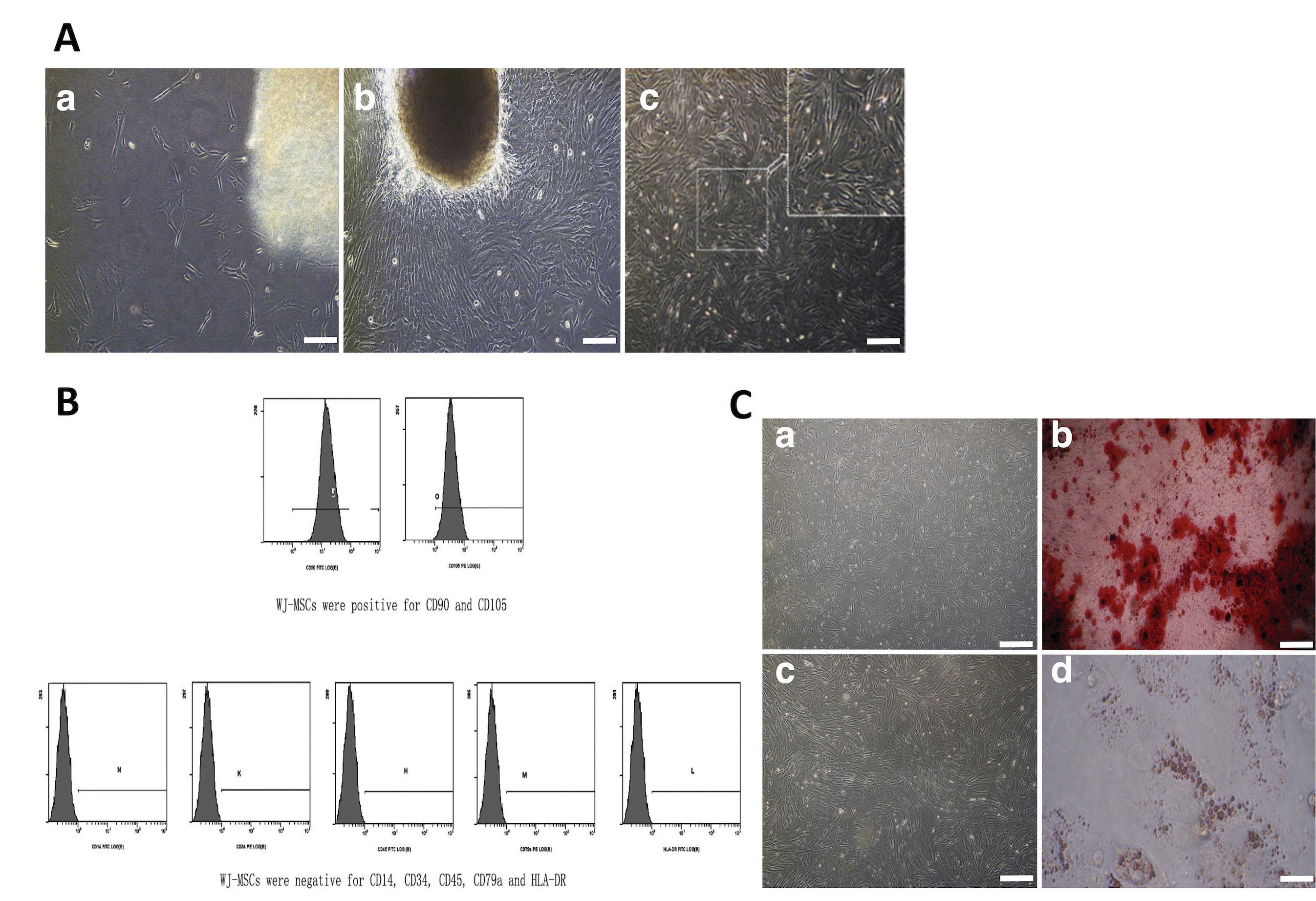

Supplement: Supplementary file 1 — showing identification of WJ-MSCs. (A) Observation of WJ-MSCs under a phase-contrast microscope. (a) Seven days after the tissues of Wharton’s jelly were plated, many triangular and spindle-shaped cells dissociated from the tissues. (b) About half a month later, these adherent cells were able to reach 80% confluence. (c) Third-generation cells exhibited a spindle shape and upon reaching confluence formed a whirlpool-like pattern. Bar represents 200 μm. (B) Surface antigens of WJ-MSCs in flow cytometry. WJ-MSCs were positive for CD90 and CD105; WJ-MSCs were negative for CD14, CD34, CD45, CD79a, and HLA-DR. Results confirmed that cells were MSCs but nonhematopoietic. (C) Differentiation potential of WJ-MSCs toward osteogenic and adipogenic lineages. Osteogenic differentiation assayed using the von Kossa procedure and adipogenic differentiation determined by formation of lipid vacuoles after induction. (a) No mineralized matrix formation found in WJ-MSCs cultured in regular growth medium. (b) Osteogenic differentiation determined by staining with Alizarin red after osteogeneic induction. (c) No lipid vacuoles found in WJ-MSCs cultured in regular medium. (d) Adipogenic differentiation detected by Oil red O staining. Bar represents 400 μm [file 13287_2017_700_MOESM1_ESM.tiff]

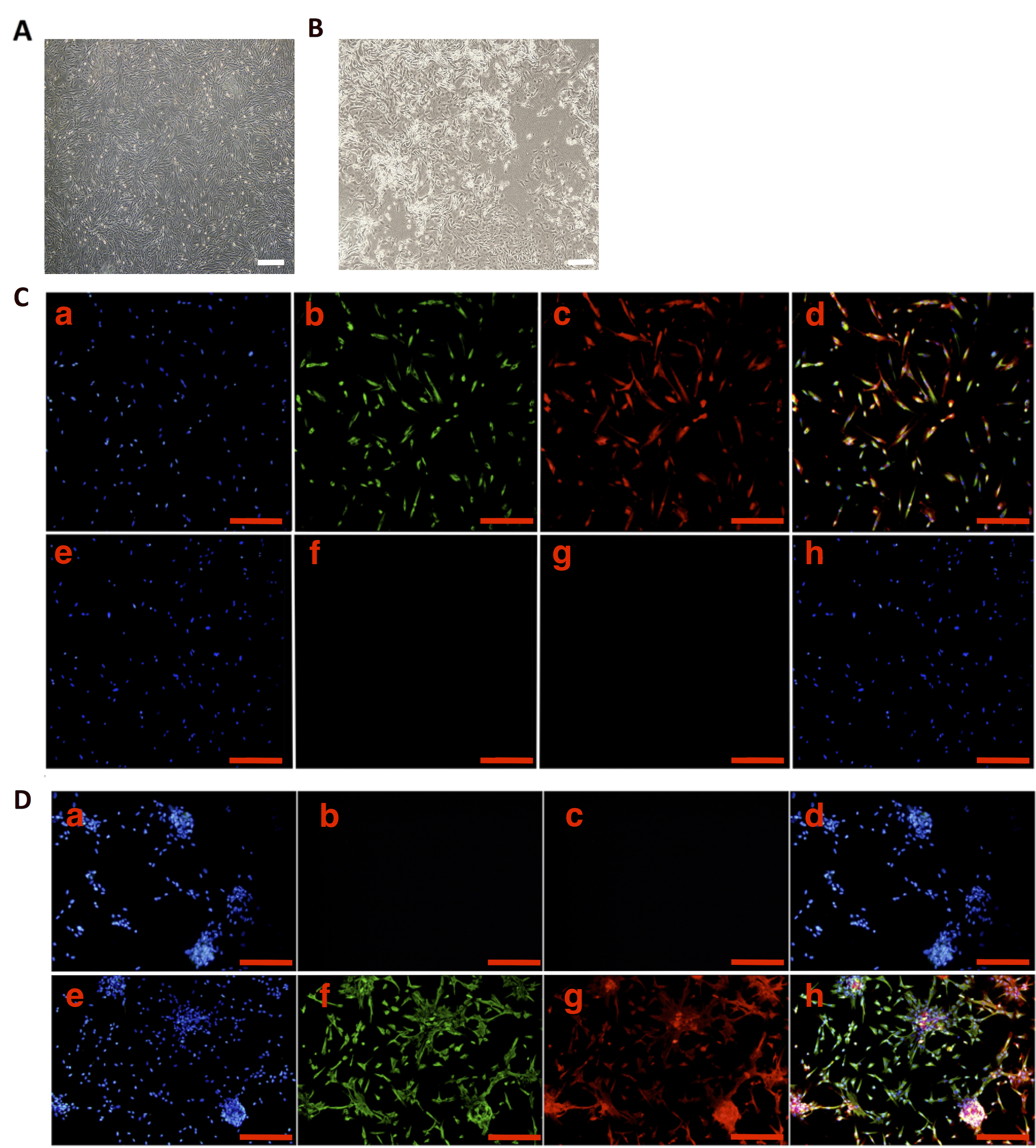

Supplement: Supplementary file 2 — showing identification of ESCs and EECs. (A) Morphological characteristics of ESCs. Bar represents 200 μm. (B) Morphological characteristics of EECs. Bar represents 200 μm. (C) Observation of ESCs after immunofluorescent staining. Results show ESCs in primary culture positively stained by vimentin and CD13 but negatively stained for cytokeratin and CD9. (a), (e) Nuclear counterstaining with Hoechst 33342. (b) ESCs positively stained by vimentin. (c) ESCs positively stained by CD13. (d) Merger of (a)–(c). (f) ESCs negatively stained by cytokeratin. (g) ESCs negatively stained by CD9. (h) Merger of (e)–(g). Bar represents 200 μm. (D) Observation of EECs after immunofluorescent staining. Results show that EECs in primary culture were positively stained by cytokeratin and CD9 but negatively stained for vimentin and CD13. (a), (e) Nucleal counterstaining with Hoechst 33342. (b) EECs negatively stained by vimentin. (c) EECs negatively stained by CD13. (d) Merger of (a)–(c). (f) EECs positively stained by cytokeratin. (g) ESCs positively stained by CD9. (h) Merger of (e)–(g). Bar represents 200 μm (TIFF 31403 kb) [file 13287_2017_700_MOESM2_ESM.tiff]
